# Impacts of Plant Protection Products on Biodiversity: Limits of Risk Assessment and Avenues to Ground Liability

**DOI:** 10.1111/risa.70129

**Published:** 2025-10-16

**Authors:** Sophie Leenhardt, Laure Mamy, Carole Barthélémy, Philippe Berny, Eve Bureau‐Point, Marie‐France Corio‐Costet, Stéphane Pesce, Isabelle Doussan

**Affiliations:** ^1^ INRAE ‐ DEPE Paris France; ^2^ INRAE ‐ ECOSYS Palaiseau France; ^3^ Aix‐Marseille Université ‐ LPED Marseille France; ^4^ VetAgro Sup Marcy‐l'Etoile France; ^5^ CNRS ‐ Centre Norbert Elias Marseille France; ^6^ INRAE ‐ SAVE Villenave d'Ornon France; ^7^ INRAE ‐ Riverly Villeurbanne France; ^8^ INRAE ‐ CREDEG Valbonne France

**Keywords:** ecotoxicity, science‐based decision‐making, pesticides, policy, regulations

## Abstract

Plant protection products (PPPs) are intended to protect plants against pests. However, they are also known to contribute unequivocally to the decline of biodiversity due to their negative impact on biological groups such as terrestrial invertebrates, birds, and amphibians. At the intersection of ecotoxicology, social sciences, and law, numerous studies address the discrepancy between the regulatory framework's objectives to protect biodiversity and the actually observed impacts of legally used PPPs. The main reasons put forward are the normalization constraints of ecotoxicity tests and the inability to anticipate effects in the current substance‐by‐substance risk assessment process given the complexity of multifactorial interactions in the ecosystems and the real conditions of PPP use. Therefore, the greater the consideration given to the systemic complexity of indirect effects, the less possible it is to quantify the contribution of a given cause, in our case, a PPP. This is a core issue in legal disputes regarding the liability of those who develop PPPs, those who use them, as well as decision‐makers who approve them. This article explores legal possibilities to better address the missing link between substance‐by‐substance assessment and authorization processes, and biodiversity protection instruments. The aim is to question the division of roles between scientific expertise, legal disputes, and public policy.

## 1 Introduction

The importance of preserving biodiversity is emphasized on a global scale (IPBES, 2019). Among multiple causes (the main ones being land and sea use changes, unsustainable direct exploitation of biological resources, climate change, chemical pollution, and invasive alien species; IPBES 2019), direct and indirect effects of plant protection products (PPPs) have been identified as significant contributors to biodiversity decline (Pesce et al. 2025). In particular, they are a major driver of population decline in common birds, terrestrial and aquatic invertebrates, and amphibians, and they impact a wide range of ecological functions (Brühl and Zaller 2019; Liess et al. 2021; Leenhardt et al. 2023; Mamy et al. 2022; Pesce et al. 2025; Sánchez‐Bayo 2021; Sánchez‐Bayo and Wyckhuys 2019). Besides other chemicals or biocides, PPPs are currently governed by specific legislations that define them as products that consist of or contain active substances, safeners or synergists, and are used to protect plants against pests (Regulation (EC) No 1107/2009 2009). Other pesticides or biocides intended for veterinary or sanitary use, for example, are not included in the scope of this definition.

Institutions such as the European Food Safety Authority (EFSA) in the European Union (EU) and the United States Environmental Protection Agency (USEPA) in the USA have set up processes for the regulatory risk assessment of PPPs. One of the main declared objectives is to authorize only PPPs that do not have unacceptable effects on the environment. However, there is a gap between, on the one hand, the regulatory framework for PPPs risk assessment and its ambitious objectives, and, on the other, scientific evidence of the adverse consequences of their use for the environment. Such inconsistency is well known and documented in the field of ecotoxicology as well as in the social sciences (Doussan et al. 2024). Moreover, it has led to a proliferation of legal disputes, particularly against governments, in view of their role in protecting biodiversity (Bailleux 2020; Van Lang 2023). This rise in legal complaints prompts a question: who is responsible, and to what extent, for the impacts of PPPs on biodiversity?

Therefore, the aim of our work is (1) to take stock of the existing knowledge on the limitations of assessing the risks of PPPs for biodiversity; (2) to question possibilities in the legal area for holding actors liable; (3) to conclude on the division of roles between scientific expertise, legal disputes, and public policy.

## 2 Limitations in Assessing the Risks of PPPs for Biodiversity

Focusing on the EU regulatory framework can be relevant with regard to risk assessment limitations, as it is the one that most strictly considers impacts on biodiversity (Chen et al. 2023; Donley 2019; Gehen et al. 2019). Regulation (EC) No 1107/2009 (2009) concerning the placing of PPPs on the market claims, on behalf of the precautionary principle, that only PPPs that do not have any harmful effect on human or animal health nor any unacceptable effects on the environment, including biodiversity and ecosystems, can be placed on the market. Over the past 15 years, this regulatory framework, supported by scientific knowledge, has enabled the withdrawal of some PPPs due to their environmental persistence and/or their proven ecotoxicity (EU Pesticides Database 2025). However, the chronic and complex ecotoxicological effects of PPPs are still often underestimated by the risk assessment procedures on which the regulatory framework is based (Doussan et al. 2024). This has led to research work in various disciplines, allowing several difficulties to be identified.

### 2.1 The Impossible Proof of Absence of Effects

Whatever the experimental strategy or the type of in situ monitoring, it is conceptually impossible to establish scientifically that a PPP has no unintended ecotoxicological effects. Indeed, the absence of observed effects, based on the biological descriptors taken into consideration, leads to two hypotheses between which no conclusion can be reached: either there are no effects, or the experimental setup is inadequate to detect effects that actually exist (e.g., on a different scale, or affecting other organisms or other parameters than those measured according to the used protocol).

This is why, at a regulatory level, Regulation (EC) No 1107/2009 (2009) uses the term “no unacceptable effects” (art. 4‐3b) rather than “no effect” on the environment. In EFSA's documents as well, risk assessment leads to conclusions such as “did not identify critical areas of concern” or “the available information does not allow firm conclusions to be drawn on this aspect of the risk assessment” (EFSA 2023a).

These conclusions are based on regulatory standardized tests carried out under a limited number of controlled conditions, enabling results to be compared (Doussan et al. 2024; Pesce et al. 2025). The tests are well‐suited to the restrictive question for which they were designed, and they are only effective under specified operating conditions. Thus, by design, any effects falling outside the test protocols are disregarded, including untested species, untested exposure durations, cumulative or interactive effects, and unexpected types of effects that lie beyond the known mode of action of the tested substance (Doussan et al. 2024; Topping et al. 2020).

### 2.2 The “Unacceptable” Level of Effects

Furthermore, ensuring the absence of “unacceptable effects” as required by European PPP regulations calls for the scientific determination of an acceptable limit of effect. Yet, this notion of acceptability is political rather than scientific (Leonelli 2021). This is particularly well illustrated by the negotiations on risk assessment guidelines for pollinators. Following alerts concerning the effects of neonicotinoids on pollinators, EFSA published guidelines for assessing such effects back in 2013 (EFSA 2013). However, due to opposition from some Member States who anticipated that the assessment methods would result in too many active substances being banned, this document was never fully endorsed by the Standing Committee on Plants, Animals, Food and Feed (SCoPAFF) despite several years of attempts. EFSA was thus asked to submit a new proposal, which was not finalized until the end of a largely negotiated process in May 2023. This document accepts a reduction in colony size following exposure to a PPP of up to 10% for honeybees (EFSA 2023b).

### 2.3 The Choice of Baseline

Another key issue at the intersection of science and politics is the choice of a baseline that reflects the state of non‐impacted biodiversity. In laboratory testing, protocols consist of a comparison of the tested effects with and without the PPP. In reality, the “without PPP” equivalent situation does not rigorously exist, due to the extent of PPP contamination, and to the diversity of parameters interfering in the contexts considered. More broadly, this issue relates to environmental law and how baseline conditions can be defined and integrated into legal frameworks. This topic was extensively explored by Craig (2015).

### 2.4 Scientific Knowledge Not Sufficiently Taken Into Account

Numerous studies in the social sciences, as well as some opinion papers in ecotoxicology, pointed to the highly incomplete nature of the scientific information considered for PPP risk assessment (Brühl and Zaller 2019; Doussan et al. 2024; Pesce et al. 2025; Rudén et al. 2017; Schäfer et al. 2019). They mention the following main reasons.

Legal normativity comes with a series of requirements to meet the criteria of stability and predictability that prevail in law. While these requirements are necessary to enable the comparison and validation of the scientific findings used in authorization decisions, they are also a major factor in the disregard of a significant proportion of knowledge. They come against the need for constant updating and renewed expertise on a case‐by‐case basis, in line with the evolution and heterogeneity of the available scientific literature, due notably to constant technological, methodological, and conceptual improvements in the field of ecotoxicology. Scientific knowledge is generally only considered in regulatory risk assessments of PPPs if it complies with internationally standardized ISO or OECD specifications (Robinson et al. 2020). Yet numerous publications point to significant shortcomings in the standardization process, particularly regarding the management of conflicts of interest, transparency, and the scientific justification of decisions (Martin 2020). Furthermore, studies conducted according to protocols other than those required by the regulation, provided they are scientifically sound, could yield complementary insights (Rudén et al. 2017; Topping et al. 2020).

The fact that regulatory risk assessment studies of PPPs are carried out by the companies that are marketing these chemicals also raises questions. On the one hand, companies must assume the financial burden of proving that the PPP complies with existing rules, and on the other hand, it makes it difficult to ensure the transparency and reliability of the results provided (Leonelli 2021). Some results highlighting undesirable effects have even been deliberately concealed (Krimsky and Gillam 2018; Mie and Rudén 2023; Robinson et al. 2020).

Additionally, some counter‐assessments conducted independently of the authorization procedure revealed the non‐systematic nature of knowledge selection for risk assessment, and exposed scientific misconduct (Goulet et al. 2023; Robinson et al. 2020).

Risk assessment processes adhere to criteria such as repeatability, comparability, predictability, and neutrality, which are key elements of the regulatory process. However, the combination of the above factors results in some of the scientifically valid knowledge being overlooked (Dedieu 2021).

### 2.5 Missing Scientific Knowledge

Even if we assume that all reliable scientific knowledge were given more consideration, it would still be impossible for science to document ex ante all the consequences of the use of a PPP on a spatiotemporal scale representative of real conditions. Biodiversity decline is a multifactorial issue, with many parameters interfering with the impacts of PPPs (Brühl and Zaller 2019; IPBES 2019).

**FIGURE 1:**
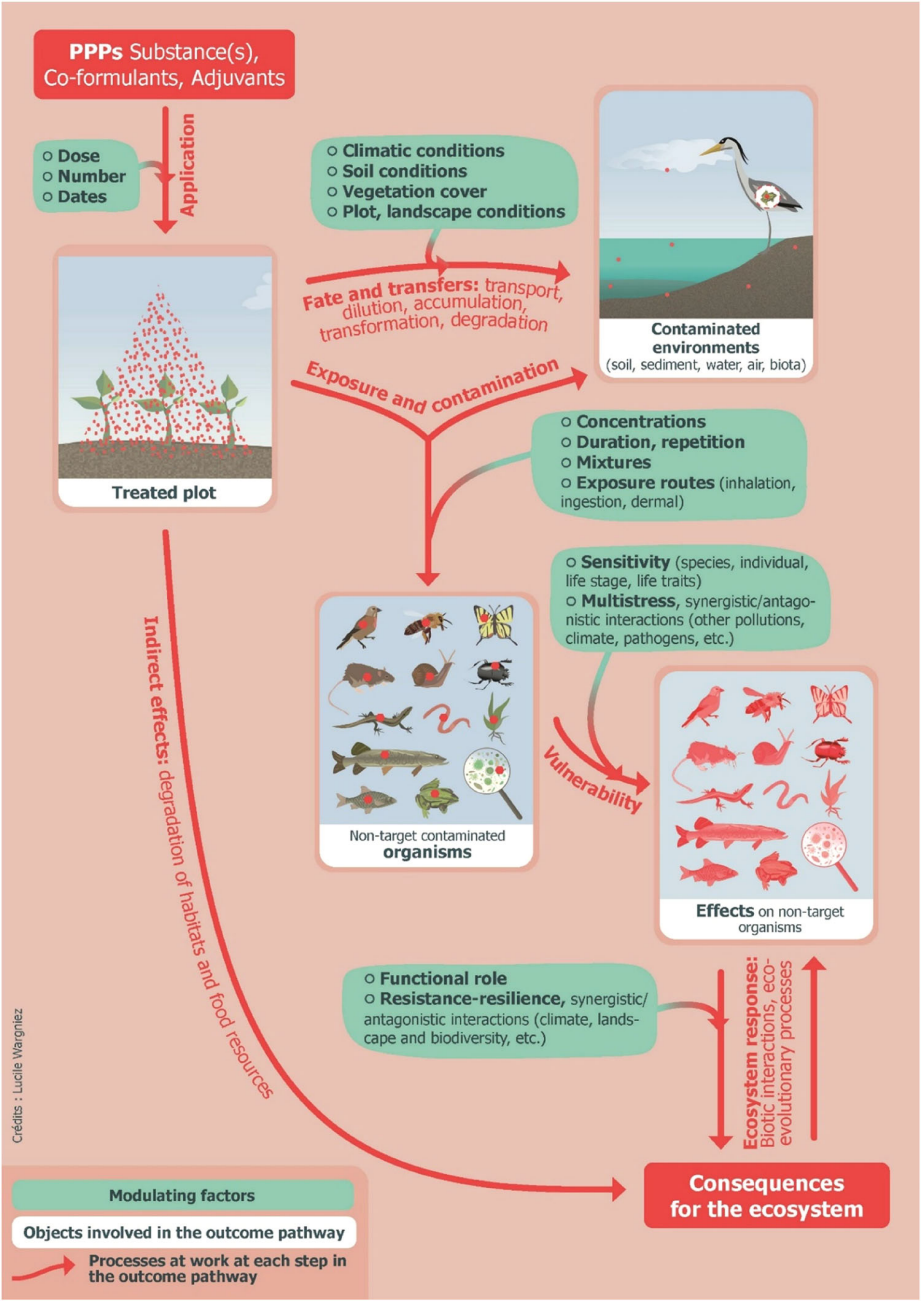
PPP's impacts on biodiversity—Objects and factors involved at each stage of the outcome pathway.

Figure 1 illustrates, through a simplified linear chain of consequences, the main scientifically analyzable stages that break down the question of the impacts of PPPs on biodiversity (Pesce et al. 2025). In reality, however, there are considerable interactions between these stages, with effects that combine and interact in a bushy rather than linear fashion. Thus, at each stage, not one but several scenarios need to be considered in relation to the multiplicity and variety of PPPs used (and the simultaneous or successive nature of their presence in the environment), of plots treated, of receiving environments and organisms exposed, as well as the transformation of the substances in the environment, and so forth. Moreover, the factors that influence the magnitude of the consequences observed at each stage depend heavily on the environmental context in a given place and time. The heterogeneity of these particular conditions, which will be encountered in real‐life situations and influence the fate and effects of each PPP, cannot be captured by a generic assessment approach. Consequently, a set of assumptions must be made during the risk assessment process. In‐depth work was carried out to narrow down the situations to be considered, primarily based on PPP properties and worst‐case scenarios. However, despite this progress, the wide range of factors influencing each stage of the outcome pathway analyzed in Figure 1 remains far from fully addressed. To account for the extent of these uncertainties, assessment factors ranging from 2 to 100 are applied to multiply the effects observed in regulatory tests (Regulation (EC) No 1107/2009, 2009; Topping et al. 2020). However, this correction only applies to the results of studies carried out on an extremely limited number of species and contexts, as required by the regulation. The species used are chosen to represent key ecological groups and functions (e.g., pollinators, decomposers, primary producers), covering both terrestrial and aquatic environments, based on practical considerations. Indeed, they must be sensitive enough to detect potential toxicity according to standardized test protocols. However, such a strategy does not consider untested effects (in particular indirect effects), cumulative pressure (e.g., cocktail effects, vulnerability to climate change, emerging diseases), or chronic exposure (Brock et al. 2016; Devos et al. 2016; Doussan et al. 2024; Levine and Borgert 2018; Martin 2016, 2020; Ockleford et al. 2018; Pesce et al. 2025; Robinson et al. 2020; Schäfer et al. 2019; Topping et al. 2020).

### 2.6 Avenues for Improvement

Due to the above‐described complexity, increasing the realism of scientific work also raises the challenge of characterizing the relationship between a PPP and biodiversity (Andrade et al. 2021; Delmas 2020). Such questions are the subject of a great deal of research aiming at improving PPP risk assessment (Doussan et al. 2024). Some studies have overcome controversies regarding the deciphering and quantification of the pressure–effect relationship (Rigal et al. 2023). In particular, work is underway to develop the Adverse Outcome Pathway (AOP) approach (Ankley et al. 2010), which is complemented by Aggregate Exposure Pathways (AEP) (Hines et al. 2018), to integrate the various stress factors and the dynamics of impacts at different levels of biological organization. The limitations of a strictly mechanistic approach are also increasingly recognized, paving the way for explanatory analyses of sets of results when estimating the weight of evidence (WoE) (LaLone et al. 2017; Suter et al. 2017) of different pieces of information. Ecological modelling, particularly at the landscape scale, is also employed to take better account of the complexity of situations encountered (Boivin and Poulsen 2017; Larras et al. 2022).

The extent of scientific knowledge considered in regulatory frameworks could be increased through two complementary dynamics: fostering, in the scientific sphere, the development and implementation of protocols complying with regulatory standards when this is methodologically relevant, and better integrating within the regulatory process the relevance of non‐standardized scientific results. However, such a move would be demanding, as non‐standardized studies require more time for assessment and a higher level of expertise, and give rise to divergence as there are no ready‐to‐use guidelines to refer to.

A posteriori observation of PPP impacts could also facilitate better management that is aligned with real environmental conditions. For example, France's phytopharmacovigilance system, which is unique in Europe (Botta et al. 2018), has been collecting and analyzing monitoring data on PPPs since 2015. This system raises alerts as early as possible on any adverse effects related to the use of these products (Agence nationale de sécurité sanitaire de l'alimentation, de l'environnement et du travail [ANSES] 2021). These observations are combined with scientific literature, and additional studies may be funded based on the resulting identified needs (Mamy et al. 2022; Volatier et al. 2019). However, although the results are intended to inform reassessment processes, they have rarely resulted in the withdrawal of authorization to date.

Finally, a compromise remains unavoidable between attributing responsibility (a substance‐by‐substance approach) and recognizing systemic effects (which cover multifactorial and indirect interactions). Despite progress in both regulation and knowledge, and as protection objectives and the precision of assessments advance, the difficulties in producing conclusive scientific evaluations become increasingly apparent. This exacerbates divisions between stakeholders.

## 3 Possibilities Browsed to Better Engage Liability

The pledge of a regulatory framework to approve only the PPPs with no unacceptable effects on the environment cannot be fulfilled based solely on risk assessment processes. The extent of such discrepancy brings discredit (Dedieu 2021; Médevielle et al. 2019) and leads to an increase in legal disputes. These disputes are based on legal principles that cover more broadly environmental matters, such as the public's right to information and justice in environmental matters, or environmental liability. These principles apply at various levels (e.g., Aarhus Convention at the international level; Directive 2003/4/EC, 2003 on public access to environmental information, and Directive 2004/35/EC, 2004 on environmental liability at the EU level; national laws). However, to successfully engage liability in legal proceedings, the following key elements are required.

### 3.1 Transparency

Access to the scientific information provided by the petitioner for a PPP marketing authorization is the first step in checking compliance with the conditions set out in Regulation (EC) No 1107/2009 (2009). Appeals have been made against marketing authorization decisions relating to the type and statistical quality of the data. For example, in France, any person with a legitimate interest in taking action can request an administrative judge to review compliance. This may result in the public decision being invalidated on the grounds of unfoundedness (Cour administrative d'appel de Lyon 2021). Regulation (EU) 2019/1381 (2019) on the transparency and sustainability of the EU risk assessment in the food chain came into force in 2021 and opened up public access to that scientific data. However, this type of appeal faces two main limitations: (1) it may be challenged by petitioners invoking the right to industrial secrecy. Nonetheless, for some years now, this right has been interpreted restrictively by case law, which, on the contrary, takes a broader view of the public's right to information on PPPs (Chearnaigh 2021; European Court of Justice 2016, 2019); (2) it is difficult to implement for organizations with limited resources, and therefore applies to only a small number of cases (Robinson et al. 2020).

### 3.2 Causality Link

The multifactorial nature of biodiversity decline makes it difficult to establish liability. While a systemic approach that takes into account the multitude of interactions is necessary to meet realistic environmental conditions, it also makes it more difficult to isolate and quantify one specific causal link. The regime provided by Directive 2004/35/CE (2004) on environmental liability expressly excludes pollution of a “diffuse character,” unless a causal link can be established between the damage and the activities of individual operators. However, in a recent decision at the French national level, the court acknowledged that biodiversity decline has multiple causes, but nevertheless found that the ecological damage resulting from the use of PPPs is well established (Cour administrative d'appel de Paris 2025). This raises the question of who should be held liable.

### 3.3 The Actor to be Held Liable

Complaints are filed at different levels, mostly by non‐governmental organizations (NGOs), given that biodiversity, the “victim”, has no means of engaging liability. On the opposite side, responsibility assignment between players such as institutional actors (agencies that assess risk, governments that grant applications), PPP producers and final users is unclear.

In theory, the authority may be held liable for authorizing PPPs that do not offer the safety guarantees required by regulations. In practice, so far, the judges’ position has remained unclear on whether marketing authorizations issued for products with “unacceptable” effects on the environment constitute a fault engaging the State's responsibility. The extent to which these shortcomings contribute to recognized ecological damage is a matter of debate. However, recently, the Administrative Court of Appeal of Paris in France recognized the State's responsibility for the damage caused to biodiversity by PPPs (Grimonprez 2023; Cour administrative d'appel de Paris 2025). The Court of Appeal holds the State liable on the basis of its shortcomings in risk assessment, which contribute to the worsening of ecological damage. This leads to the State's obligation to modify the risk assessment procedure and reconsider certain marketing authorizations. But, in the same ruling, the court dismissed any obligation to restore damaged biodiversity on the grounds that the applicant associations had not specified the necessary measures or their likely cost if reparation was not possible.

Regarding PPP producers, existing cases mainly focus on the impact on human health rather than the environment. Additionally, the producer may invoke non‐compliant conditions of use for the application of PPPs at the farmer's level (e.g., incorrect dosages or application periods). In this case, the PPP user is like a screen in the chain of responsibility between the damage and the producer.

Conversely, the user may more easily incur civil and criminal liability in the event of one‐off damage (e.g., pollution of a watercourse or death of domestic bees), as demonstrated in national‐level trials, for example, in France (Cour administrative d'appel de Marseille 2011; Cour d'appel de Rennes 2015).

### 3.4 Avenues for Improvement

In view of the difficulties of enforcing liability, a mutualized assumption of responsibility for the harmful consequences of PPP use could be sought. Avenues can be identified in the tax area, in line with the “polluter pays” principle. For example, PPP distributors in France pay taxes for diffuse pollution, which are modulated according to the quantity sold, the toxicity, and the hazard of the PPP. However, these taxes do not cover the cost of cleaning up drinking water (Alliot et al. 2022; Bommelaer and Devaux 2011) or the impact on biodiversity. A solution could be devised along the lines of existing compensation funds, such as those set up for maritime oil pollution (https://iopcfunds.org/). In France, such a fund is dedicated to the compensation of PPP victims not covered by occupational disease insurance (Articles L. 491‐1 to L. 491–7, French Social security code), based on the principle of national solidarity and the State's liability for issuing and maintaining marketing authorizations for PPPs. However, as with any compensation regime, this principle only provides for the funding for restoration measures. Given the irreversible nature of certain biodiversity degradation, preventing such damage remains paramount.

In addition, the diffuse nature of the effects of PPPs opens up a research field concerning the identification of a combination of measures that can restore the ecosystem functions affected by the use of PPPs, and the quantification of the cost of these measures, as required by the Administrative Court of Appeal of Paris (Cour administrative d'appel de Paris 2025).

## 4 Conclusion

Well‐established knowledge regarding the impacts of PPPs on biodiversity shows that, while the current substance‐by‐substance approach of risk assessment is necessary, it remains insufficient to prevent unacceptable effects on biodiversity. Though scientific research continues to improve our understanding of the effects of PPPs on biodiversity, it is essential to recognize that the part of it considered for decision‐making covers only partially the issue to be addressed. The same limitations hamper the filing of complaints, which rarely result in the responsible parties being held accountable for the damage caused. However, there has been some movement on this issue with the French government recently being condemned for not taking sufficient account of the latest scientific findings, particularly, with regard to the effects on non‐target species (Cour administrative d'appel de Paris 2025). On the one hand, the judge has confirmed the liability and fault, and ordered a revision of the assessments, including for products that have already been authorized. On the other hand, it dismissed any obligation to restore damaged biodiversity on the grounds that measures to restore the affected biodiversity or their likely cost if reparation was not possible, were not identified.

The gap between the objective of protection enshrined in law and observed impacts, as well as the multifaceted nature of causes and liabilities, is at the heart of the debate on PPP pollution. This has led to a polarization of views among assessment agencies with one extreme being the complete rejection of scientific expertise (Dedieu 2021; Médevielle et al. 2019) and the other being the expectation that scientific results will be directly applicable to political decision‐making (Hamlyn 2017). The notion of science‐based decision‐making is indeed often invoked as a guarantee of political virtue. However, the limitation of the findings considered in such procedures leads to their denunciation in legal disputes.

Our analysis questions the division of roles between scientific expertise, legal disputes, and public policy. In order to fulfil the liability process, further research is required to identify the measures that could be implemented to restore biodiversity affected by PPPs, and to determine their cost.

There is also room to consider scientific knowledge more broadly. Questions that are scientifically uncertain or incomplete must clearly be posed as part of an open and informed political debate, with regard to the ethical and political issues associated with PPPs (Demortain 2021; Kleinman and Suryanarayanan 2013, 2020; Möhring et al. 2020; Prete 2013). Beyond the standardized processes of risk assessment, scientific findings can contribute to ethical and political debates concerning the consequences of greater or lesser dependence on PPPs. This would shed light on all aspects of the trade‐offs associated with using PPPs and allow open consideration of the choices to be made and responsibilities involved.

## Data Availability

Data sharing not applicable to this article as no datasets were generated or analyzed during the current study
